# How much is too much? The effects of information quantity on crowdfunding performance

**DOI:** 10.1371/journal.pone.0192012

**Published:** 2018-03-14

**Authors:** Naomi Moy, Ho Fai Chan, Benno Torgler

**Affiliations:** 1 School of Economics & Finance, Faculty of Business, Queensland University of Technology, Brisbane, Queensland, Australia; 2 Center for Research in Economics, Management and the Arts (CREMA), Zurich, Switzerland; Consejo Nacional de Investigaciones Cientificas y Tecnicas, ARGENTINA

## Abstract

We explore the effects of the quantity of information on the tendency to contribute to crowdfunding campaigns. Using the crowdfunding platform Kickstarter, we analyze the campaign descriptions and the performance of over 70,000 projects. We look empirically at the effect of information quantity (word count) on funding success (as measure by amount raised and number of backers). Within this empirical approach, we test whether an excessive amount of information will affect funding success. To do so, we test for the non-linearity (quadratic) effect of our independent variable (word count) using regression analysis. Consistent with the hypothesis that excess information will negatively affect funds raised and number of contributors, we observe a consistent U-shaped relationship between campaign text length and overall success which suggest that an optimal number of words exists within crowdfunding texts and that going over this point will reduce a project’s chance of fundraising success.

## Introduction

… in an information-rich world, the wealth of information means a dearth of something else: a scarcity of whatever it is that information consumes. What information consumes is rather obvious: it consumes the attention of its recipients. Hence a wealth of information creates a poverty of attention and a need to allocate that attention efficiently among the overabundance of information sources that might consume it.

Herbert A. Simon (1971, pp. 40–41)

It has often been said,

There’s so much to be read,

You never can cram

All those words in your head.

So the writer who breeds

More words than he needs

Is making a chore

For the reader who reads.

That’s why my belief is

The briefer the brief is,

The greater the sigh

Of the reader’s relief is.

And that’s why your books

Have such power and strength.

You publish with shorth!

(Shorth is better than length.)

Theodor Seuss Geisel (Dr. Seuss)

Lo bueno, si breve, dos veces bueno.

Baltasar Gracián

Information gathering is a crucial part of both decision-making and problem-solving, whether it be pedestrians checking for approaching traffic before crossing a road or consumers reading hotel reviews before making a reservation. Such efforts are made in the belief that having too little information could result in negative outcomes, such as a severe traffic accident or a disastrous hotel stay. When information is overabundant, on the other hand, individuals cannot process every single datum even though doing so would probably lead to a better outcome (e.g., a higher quality decision). Likewise, when confronted with an information surplus like a lengthy text, they find it difficult to distinguish between relevant and unnecessary data. Rather, because the cognitive costs of acquiring all relevant information outweigh the potential benefits [1], they tend to rely on heuristic or reasoning based shortcuts to reach decisions. These observations have led the developing field of attention economics to treat human attention as a scarce resource [2], a limiting factor in information consumption.

To throw more light on this relationship between the amount of information provided and decision-making, this paper examines it in the context of the crowdfunding platform Kickstarter, which seeks public financial support for innovative projects and ideas. Because potential contributors rely on project description content to inform their pledging decision, it is important to understand the role of information quantity in investment behaviour. To this end, our analysis is guided by one primary research question: Does the amount of information provided by the creator influence the funding outcome? Using a large empirical dataset from Kickstarter.com that encompasses almost 80,000 projects, we identify an inverted U-shaped relation between project description length and number of funders or amount raised.

## Text length and information

Information overload occurs when individuals receive too much information and reach a point at which they can no longer process information [1], [3]. In effect, too much information becomes too much of a good thing [4]. This overabundance is evidenced by individual performance during decision-making, which steadily improves as information is added but then declines as data input becomes too much and leads to information overload (see [3], for an overview). Providing too much textual information, therefore, and thereby increasing text length could (unintentionally) have a negative effect on individual contributor decisions. It may, for example, lead to feelings of stress, confusion, pressure, anxiety, or low motivation ([3], p. 328). Naturally, the propensity to ignore lengthy informative texts is closely related to individual attention spans. Moreover, the problems associated with attention span can be further exacerbated by comprehensibility of certain text sections being too dependent on a clear understanding of preceding parts. As well illustrated by the “TL;DR” (“too long, didn’t read”) notation on very long articles, readers can usually gauge the level of effort required to digest information through such cues as the number of pages or thickness of a document. Academic journal articles, for example, tend to be around 20 pages long, while many non-fiction books are no longer than 300 pages and online news articles are growing progressively becoming shorter [5].

The effect of changing writing style in terms of text length is well investigated in various disciplines. For example, various studies in the informetrics and scientometrics literature report a significantly positive relation between scientific article length and citation outcome (see [6], [7], [8], [9], [10]). On the other hand, short and succinct abstracts are more likely to increase citations than longer abstracts [11]. A large body of survey research literature is similarly devoted to studying the link between response rates and questionnaire length. Whereas shorter questionnaires tend to increase both response quality and response rate [12], [13], [14], (also see [15] for an overview), a distinct U-shaped relation is reported between response rate and questionnaire length [16]. In other words, although shorter questionnaires elicit the greatest response rate, the longest questionnaires do not necessarily have the lowest response rate.

In a consumer setting such as exhibit labels in a museum or health claims on foodstuffs, text length can affect individual attention. For example, whereas the longer the text, the less likely it is to be read or understood (see [17], [18], [19], [20]), the longer the online review for consumer products, the more positively it is correlated with purchasing behaviour. For instance, [21] examines the effect of the average length of online book reviews on book sales on Amazon.com and Barnesandnoble.com and [22] studies the relationship between comments length and perceived helpfulness of online reviews on Yelp.com. Research concludes that in the absence of information overload which would diminish attentiveness and lower decision quality [1], [23], [24], [25], [26], longer commentaries increase the level of perceived helpfulness and increase sales [21], [22]. In sum, the length of a body of text (i.e., number of words or pages) influences various types of success, including item sales, decision quality, or article citation.

## Crowdfunding: Kickstarter

Building upon the earlier literature, our analysis assesses the effects of text length on success in an entrepreneurial setting using data from the crowdfunding website Kickstarter, which links innovators with individuals who are willing to contribute funds in exchange for physical (product) and/or non-physical (gratitude) rewards. To convince individuals to contribute to their campaigns, creators of the innovation must pitch their idea using text, which may be supported by images and videos. The amount of information used within each medium is important given Kickstarter’s all-or-nothing funding model (i.e., a project creator receives no money if the funding goal is not reached). Because both the descriptive text and outcome are discernible in this setting, we are able to observe how word number deters or increases the number of monetary contributions to a project. Even more important, by holding most things equal in outcome creation (same goal, platform, possibilities, and restrictions), Kickstarter campaigns provide a controlled setting that is equivalent to a real-world laboratory. Kickstarter descriptors are thus ideal texts for study. In our case, we anticipate an inverted U-shaped relation between project text length and funding success; that is, that an overabundance of information, quantified by number of words, will decrease the amount raised and the number of project contributors. No previous studies explore non-linearity [27], [28], [29], [30].

## Data and methodology

### Dataset

**Fig 1:**
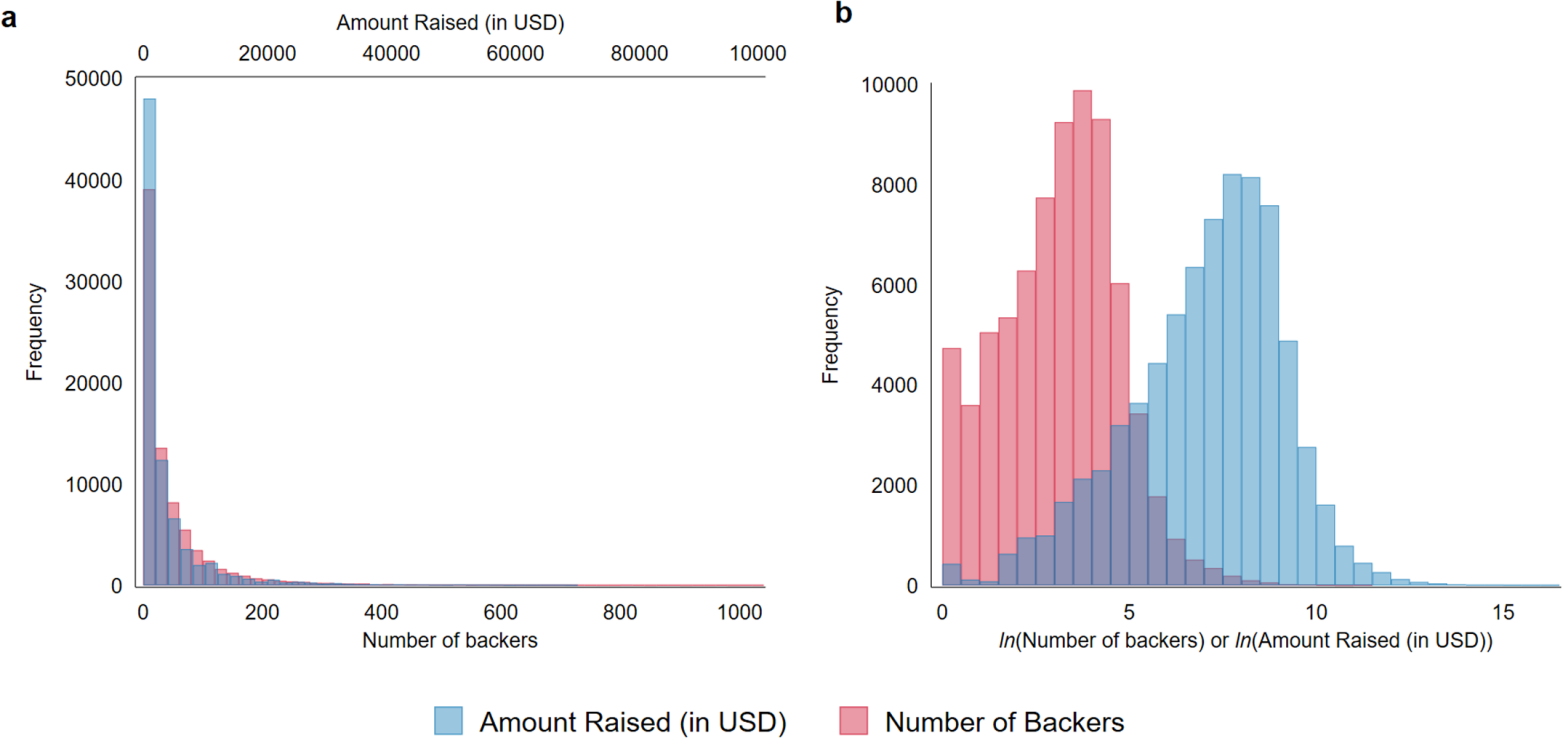
Distribution of amount raised and the number of backers, (a) original and (b) log transformed. The right-hand tail (top 99 percentile) of the distributions in (a) was not shown for the purposes of visualization.

The dataset for this study was obtained from a GitHub project developed by [31]. The raw data was collected by [31] from Kickstarter using Python, in line with previous research using data from crowdfunding websites. For example, [30] and [32] developed customized computer scripts which took daily snapshots of live campaigns on Kickstarter, [32], [33] and [34] also deployed web crawlers to extract project information from Kickstarter. Our dataset comprises detailed information on all Kickstarter campaigns from 21^st^ April 2009 to 6^th^ May 2013, for a total of 87,260 projects. Each observation records project-related information, such as outcome of the funding campaign in terms of the number of ‘backers’ (individuals who supported the campaign financially), and the amount of funds raised, full text of the campaign description, main category and sub-category of the project, funding goal, project’s geographic location, campaign launch date and end date, and project and creator identifier. We removed 3,868 projects that were live at the date of the data collection. Furthermore, we also removed 1,505 cancelled and suspended projects, or if the project description contained less than three words. The content of these projects were manually inspected, some examples of description are: “Sorry, project withdrawn!”, “Removed, for now.”, “Video to come.”. The number of projects with 1, 2 and 3 words are 126, 12 and 18, respectively. The resulting dataset spans from 21^st^ April 2009 to 29^th^ April 2013, and contains 81,892 projects. Although [34] excludes projects with over 2.5 million dollars or lower than 10 cent fundraising goals as non-serious, we retain them without any alteration to our results. For the dependent variable, we use the overall amount raised (in U.S. dollars) and the number of funding contributors to measure fundraising success of a project. These two variables provide an indication of the level of financial success and popularity of a project. When performing visual inspection of the data, we observed that the distribution of both of our dependent variables seems to follow an exponential function (see Fig 1). As a result, we apply natural log transformation to both dependent variables for our analysis.

**Fig 2:**
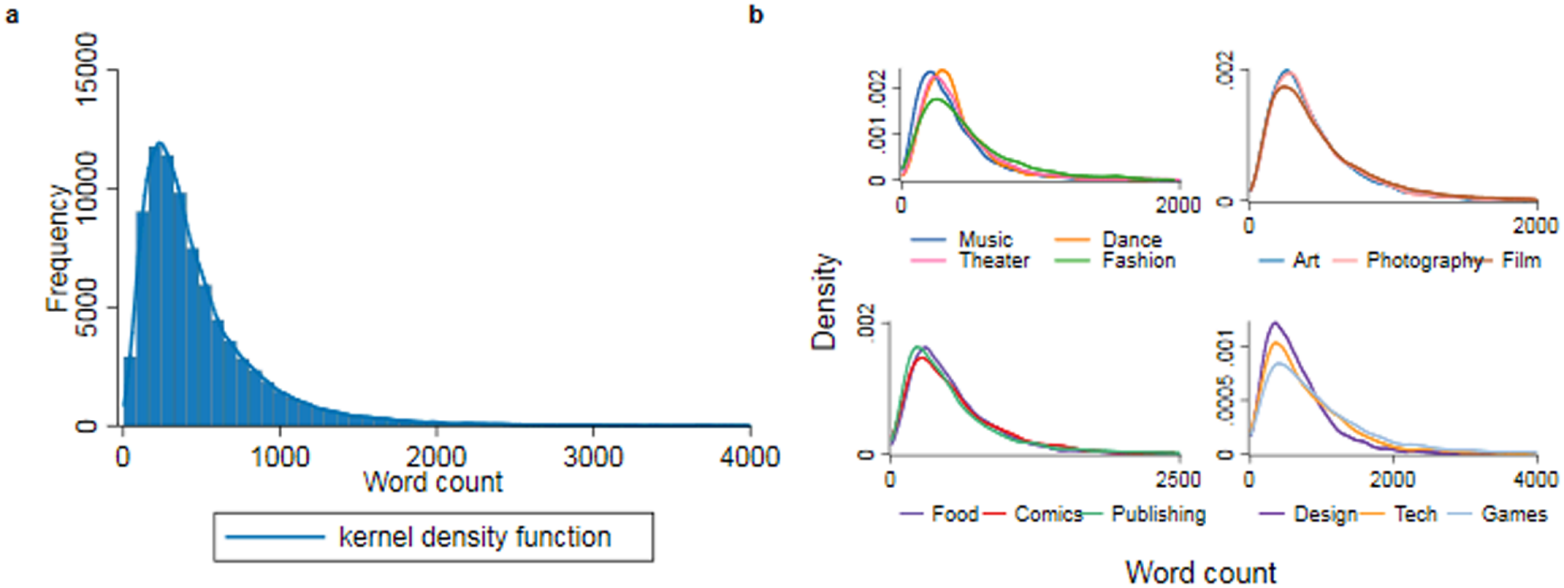
Distribution of the total word count across projects (a) and by category (b). The right-hand tail of each distribution is truncated for the purposes of visualization.

The total word count of the description of the Kickstarter project measures the key independent variable of information quantity provided by the creator. A typical Kickstarter project contains, on average, around 500 words (with a standard deviation of 466 words). It is worth noting that the distribution of project word counts varies across project categories, for example, projects in *technology* include more words than *art* projects, possibly due to the need to explain the technical aspect of the product in the former category. The summary statistics for the project description word counts are given by category in Table 1. Descriptions of *game*, *technology*, and *design* projects are longest (634–938 words on average), and descriptions of *music*, *dance*, and *theatre* projects are shortest (351–398 words on average). Additionally, as is typical for Internet-mediated texts such as emails, eforums, and Wikipedia articles [35], the distribution of description word count is right-skewed (see Fig 2). It is also notable that two projects in the publishing category provide sample chapters totaling over 10,000 words. To eliminate the possibility that these outliers, although genuine, might seriously affect the estimates of the non-linear relation between text length and funding outcomes, we censor (winsorize) the word count variable at the top 99th percentile in each category (see Table 1). In other words, we replace the value of the longest 1% of projects with the value of the 99^th^ percentile of the sample.

**Table 1:** Summary statistics of non-censored word count.

Category	N	Mean	SD	Min.	99^th^ percentile	Max.
Music	18,909	352.78	276.66	4	1353	4078
Dance	1,146	393.84	297.79	4	1424	4112
Theatre	4,003	400.10	285.71	5	1567	2572
Art	7,518	447.46	354.18	4	1761	5535
Photography	2,565	451.38	338.94	6	1766	4082
Fashion	2,597	463.11	355.57	4	1633	3275
Film & Video	21,538	498.98	418.53	4	2084	5361
Food	2,973	531.99	400.60	5	1976	4975
Publishing	9,237	547.88	666.95	4	2528	32135
Comics	2,190	569.24	486.02	9	2402	5137
Design	3,317	638.84	443.23	11	2167	4469
Technology	1,762	780.99	610.68	8	2907	5062
Games	4,137	954.48	784.61	5	3986	5370
Total	81,892	494.90	465.61	4	2230	32135

*Note*: For analysis we censored the word count variable at the 99^th^ percentile for each category.

### Description edits and potential endogeneity bias

Although the goal and project deadline cannot be edited after launch, and no information can be modified after the campaign has ended, during the actual fundraising period, project creators can amend certain project details (e.g., the text, images, and/or videos in the description) or add additional rewards. Kickstarter also provides them with a separate ‘Project update’ tab through which to communicate with (potential) backers and provide updated information. These amendments, which unfortunately are not documented, create the potential for endogeneity bias from causality issues; for example, a creator may urge potential backers to contribute as the deadline approaches or overfunded projects may offer additional rewards to attract more backers. We therefore draw on two earlier studies that assess the dynamic effect of such amendments and their possible impact on success.

In the first study, [36] examine a smaller sample of 19,299 Kickstarter projects, 64% of which underwent no edits throughout the entirety of the project. The vast majority of edited projects were only amended once or twice, most often in the first few days of the campaign. The authors do report an increased number of edits towards the project’s end, which they attribute to creators either showing appreciation for backers as the goal is being reached or urging more contributions when success seems near. However, they provide no statistical evidence for these claims. Nor do they find any statistically significant relationship between their measure of edit size (extent) and project success.

The second study [37], focuses specifically on updates from the creator via the update tab before the campaign outcome was determined. Of the 8,529 projects studied, 58.6% had at least one update and one update was significant in increasing the chances of success. To identify the frequency of project types, the authors classify the updates into seven latent Dirichlet allocation (LDA) categories. In order of most to least frequent, these are *social promotion*, *progress report*, *new content*, *reminder*, *answer question*, *new reward*, and *appreciation*. Of these, reminder, progress report, and new reward updates are the most influential in predicting project success. In our study, we account for potential endogeneity bias by using cues similar to those employed by [37] to identify edited projects. For example, we apply the label “progress report” to any description containing “*ve reached” phrases and the label “reminder” to any containing “days to go” type phrases (see S1 Table for the complete list). If, however, the description contains the word ‘update’ or ‘UPDATE’ (case sensitive) but none of the search terms intimating progress report or reminder, we classify it as a general edit that might provide information on new content or new reward. We thus code our edit indicators as no edit, general edit, reminder edit, or progress edit.

In the study by [36], they identified 6,998 (36.26%) edited projects, with a large portion only having minor amendments (e.g., corrected typos). In our case, a significant proportion of the number of projects show no identifiable amendments, however, 6,478 projects (7.91%) have been edited. We find that 72.95% of the projects with an edited description and just over 80% of the projects with a reminder or progress edit achieved funding (see Table 2). This result echoes findings from [37] that a reminder had a stronger effect on achieving success, followed by progress report, and general update (new content or reward).

**Table 2:** Summary statistics of identified edits.

	Total	Successful	Unsuccessful	Percentage successful
No edit	75414	33236	42178	44.07
General	3733	2461	1272	65.93
Reminder	592	481	111	81.25
Progress	2153	1784	369	82.86
Total (edit)	6478	4726	1752	72.95

*Notes*: 220 progress projects had been identified with a reminder *and* progress edit. They are coded as a progress given the achievement of the goal would occur after the reminder.

### Controls

As control variables we consider characteristics of the project and its creator as well as external factors within the crowdfunding platform. Project and creator characteristics include the category of the project, funding goal, funding duration (and its squared term), geographic location (latitude and longitude), and creator’s experience (using the number of existing or previous projects by the same creator). To control for the effect of change in the description text during the campaign, we include three dummy variables of each type of identified project edits, i.e., general, reminder and progress edit. Furthermore, we also consider the level of competition using the average number of projects in the same sub-category during the project campaign. Table 3 presents the descriptive statistics of the variables used in the empirical analysis.

**Table 3:** Descriptive statistics.

	Mean	SD	Min.	Max.
Amount raised (in USD)	6423.31	68773.82	0	10300000
Number of backers	88.57	843.28	0	91585
Description word count	494.90	465.61	4	32135
Funding goal (in USD)	15276.92	221083.80	0.01	21500000
Duration (in days)	37.42	16.03	1	91.96
Latitude	37.25	9.55	-54.79	78.22
Longitude	-86.62	36.66	-176.66	178.42
Project #	1.24	1.84	1	79
Avg. # project in subcategory	120.58	85.46	1	467.63
Update dummy	0.46	0.50	0	1
U.S. dummy	0.94	0.25	0	1

*Note*: Number of observations equals to 81,892.

### Model

Using the following model (1), we test the hypothesis in which excessive amount of information can affect funding success (non-linearity of word counts):

$$Y_{ik}=\beta_{0}+\beta_{1}TC_{ik}+\beta_{2}TC_{ik}^{2}+\gamma P_{ik}+\lambda C_{ik}+\rho E_{ik}+\varepsilon_{ik}$$ (1)

where,

*Y*_*ik*_ Outcome: amount raised or number of backers of the *i*th project of creator *k*

*TC_ik_* *Total word count*

$TC_{ik}^{2}$ *Total word count*, squared

***P***_***ik***_ Project characteristics (*goal*, *category*, *duration*, *duration*^*2*^, *latitude*, *longitude*, *edits dummies*).

***C***_***ik***_ Creator experience (number of existing or previous projects for the creator)

***E***_***ik***_ External characteristic, competition (average number of sub-category competitors during the campaign)

*ε*_*ik*_ Error term

## Results

**Fig 3:**
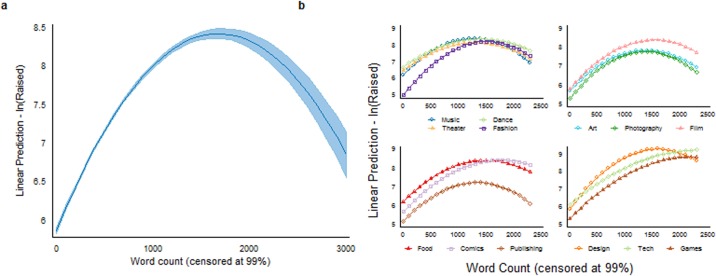
Effects of project description length (word count) on fund raised (in log). Part (a) depicts the results from specification (3) in Table 4. There exists a concave (inverted U-shape) relationship between the description length and fundraised; increasing the length of the project description increases the funding until it reaches the optimal point, (~1681.77 words), after which the amount raised is negatively affected and starts to decline. Part (b) reports the outcomes for the same specification by project category, as demonstrated in Table 5. The concave relationship is visual in all categories, but the optimal length point varies.

**Fig 4:**
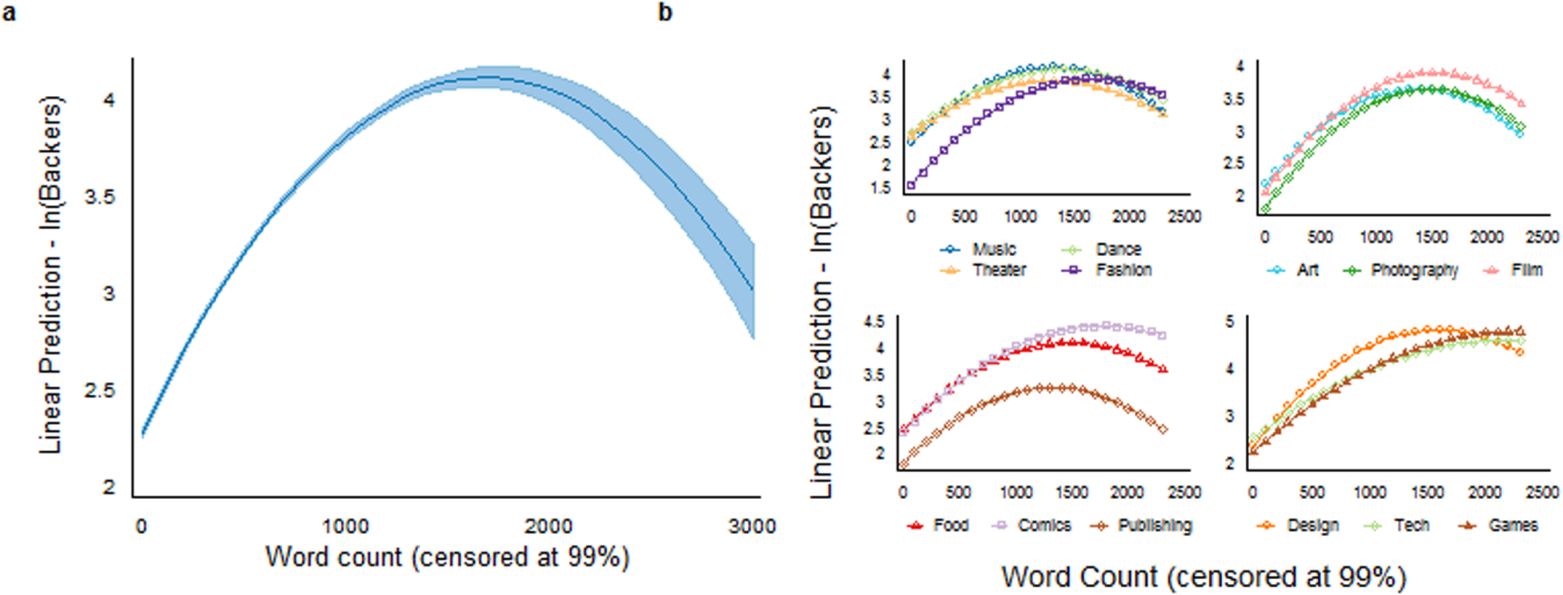
Effects of project description length (word count) on the number of contributors (in log). Part (a) depicts the results from specification (6) in Table 4. Similar to Fig 3, there is an inverted U-shape between project description length and number of backers (contributors); the optimal length point is approximately 1689.21 words. Part (b) reports the outcomes for the same specification by project category, as demonstrated in Table 6. A similar pattern was found in all categories, but the optimal length point varies.

Our estimations use a multivariate OLS regression of funds raised and number of backers contributing (in logs) on total word count of the description (as shown in Table 4). We include both the linear and squared term of word count to assess the potential non-linear relationship with the outcome variables. For all specifications, we cluster the standard errors over project creator level. Across all specifications in Table 4, the coefficient for the linear (quadratic) term of word count is positively (negatively) significant at the 1% level. This finding suggests that having more words in the project description increases both the overall amount raised and the number of contributors but with a diminishing or even negative effect once the text becomes too long. The main effects are robust to controls for project/creator characteristics and external factors. In specifications (3) and (6), we further control for potential description edits (*Edits*) and exclude projects outside the U.S. (approximately 7% of the total). Holding all other factors constant, the linear term of the word count is positive and the squared term is negative, while the coefficients are statistically significant at the 1% level. This outcome indicates a robust inverted U-shaped relation between text length and overall funding success, as illustrated in part (a) of Figs 3 and 4. For example, based on estimates from specification (3) and (6), adding 100 words to the description would increase the amount raised and number of backers of a 1000-word project by approximately 0.111% and 0.085%, respectively. On the other hand, if a project with a lengthy description (say 2000 words) added an additional 100 words, it would decrease its funds raised and number of contributors by 0.069% and 0.042% respectively. Overall, around 2.3% of the projects exceeded the optimal length of about 1,700 words.

**Table 4:** Multivariate analysis of description length (word count censored at 99%).

	*ln*(Raised)	*ln*(Raised)	*ln*(Raised)	*ln*(Backers)	*ln*(Backers)	*ln*(Backers)
	(1)	(2)	(3)^^^	(4)	(5)	(6)^^^
Word count	.0031***	.0032***	.003***	.0023***	.0024***	.0022***
	(55.12)	(58.42)	(52.72)	(50.81)	(53.95)	(48.03)
Word count^2^	-8.7e-07***	-9.1e-07***	-9.0e-07***	-6.0e-07***	-6.4e-07***	-6.4e-07***
	(-28.25)	(-29.87)	(-28.77)	(-23.47)	(-26.14)	(-25.38)
Category						
Comics		.023	-.025		.33***	.26***
		(0.44)	(-0.45)		(7.58)	(5.98)
Dance		.74***	.81***		.48***	.52***
		(14.74)	(15.81)		(12.57)	(13.23)
Design		.65***	.67***		.61***	.6***
		(14.23)	(13.94)		(15.95)	(15.18)
Fashion		-.36***	-.3***		-.38***	-.35***
		(-7.34)	(-5.99)		(-10.00)	(-9.04)
Film & video		.13***	.18***		-.043*	-.023
		(4.01)	(5.31)		(-1.81)	(-0.91)
Food		.36***	.41***		.26***	.29***
		(8.11)	(9.03)		(7.60)	(8.18)
Games		-.14***	-.17***		.28***	.21***
		(-2.72)	(-3.15)		(6.85)	(5.02)
Music		.53***	.55***		.42***	.42***
		(18.30)	(18.23)		(18.95)	(18.22)
Photography		-.24***	-.24***		-.22***	-.22***
		(-4.88)	(-4.57)		(-5.97)	(-5.70)
Publishing		-.62***	-.6***		-.4***	-.39***
		(-18.34)	(-16.81)		(-15.26)	(-14.32)
Technology		.42***	.42***		.38***	.36***
		(7.00)	(6.81)		(8.08)	(7.49)
Theatre		.53***	.57***		.35***	.36***
		(14.29)	(14.77)		(12.11)	(12.28)
Goal ($)		1.3e-07	6.2e-08		4.9e-08	1.3e-09
		(1.64)	(0.87)		(1.10)	(0.04)
Duration (days)		.014***	.013***		.0061***	.0053***
		(7.56)	(6.76)		(4.24)	(3.61)
Duration^2^		-.00018***	-.00017***		-.00012***	-.00011***
		(-9.67)	(-8.70)		(-8.33)	(-7.43)
Latitude		.0032***	.014***		.006***	.014***
		(3.95)	(12.24)		(9.56)	(15.89)
Longitude		.00032	.0011***		.0008***	.0016***
		(1.53)	(3.16)		(4.83)	(5.67)
Project #		-.017*	-.019**		-.01	-.012
		(-1.91)	(-2.17)		(-0.67)	(-0.82)
Avg. # project in sub-category		.00063***	.00052***		.00038***	.00032***
		(6.14)	(4.93)		(4.52)	(3.68)
Edits						
General edit			.89***			.85***
			(26.12)			(29.74)
Reminder			1.2***			1.1***
			(17.92)			(18.97)
Progress			1***			.98***
			(26.78)			(28.96)
N (Obs.)	74665	74665	69754	74665	74665	69754
N (Cluster)	66999	66999	62527	66999	66999	62527
*R*^*2*^	0.100	0.138	0.154	0.100	0.141	0.163

This table represents the regression results estimating the relationship between the amount raised or the number of backers with the amount of information provided by creators. The project description’s word count (Word Count), quadratic of the word count (Word Count^2^), indicator for the project category classified on Kickstarter (Category), overall goal amount of the project (Goal), the duration of the project (Duration), quadratic of the duration (Duration^2^), geographic location of the project (Latitude, Longitude), project number for the creator e.g. first or second project (Project #), average number of competitors in the project’s subcategory during the project campaign (Avg. # project in sub-category), and the indicator for edits (Edits). For robustness of our results, we conducted the regressions with the original word count variable (non-winzorised), and our results remain robust.

*Notes*: *t*-statistics are in parentheses. The reference groups for project category and edits are *art* and *no edit*, respectively.

^ Projects with a U.S. location only.

*, **, and *** designate statistical significance at the 10%, 5%, and 1% levels, respectively

Because the main effect might vary to different degrees among the various project categories, we extend our analysis by examining each category individually using the same specification structures (see (3) and (6)). As Tables 5 and 6 show, the primary finding of an inverted U-shape is evident in each category (statistically significant at a 1% level); however, the optimal length point varies over categories (see Figs 3b and 4b). The same pattern also emerges within each sub-category (see S1 and S2 Figs), with the exception being *electronic music* whose coefficient of the quadratic term is not statistically significant.

**Table 5:** Multivariate analysis of description length (word count censored at 99%) on *ln*(Raised).

	Art	Comics	Dance	Design	Fashion	Film & Video	Food	Games	Music	Photography	Publishing	Technology	Theatre
	(7)	(8)	(9)	(10)	(11)	(12)	(13)	(14)	(15)	(16)	(17)	(18)	(19)
Word count	.0031***	.0031***	.0024***	.0043***	.0043***	.0034***	.0029***	.0032***	.0035***	.0036***	.0031******	.0026***	.0026***
	(17.30)	(10.07)	(5.40)	(15.40)	(13.29)	(29.40)	(10.47)	(13.44)	(26.38)	(10.05)	(18.34)	(7.16)	(9.76)
Word count^2^	-1.1e-06***	-8.8e-07***	-8.7e-07***	-1.3e-06***	-1.4e-06***	-1.1e-06***	-9.8e-07***	-7.2e-07***	-1.4e-06***	-1.3e-06***	-1.2e-06***	-5.6e-07***	-9.8e-07***
	(-11.13)	(-5.60)	(-3.28)	(-9.81)	(-8.16)	(-17.60)	(-6.59)	(-7.46)	(-14.24)	(-6.25)	(-13.52)	(-3.48)	(-5.68)
N (Obs.)	6283	1994	1025	2924	2180	17678	2770	3596	16736	1951	7490	1527	3600
N (Cluster)	5785	1663	915	2623	2054	16186	2612	2982	15580	1832	6967	1428	3218
*R*^2^	0.112	0.181	0.088	0.232	0.191	0.133	0.119	0.305	0.105	0.137	0.111	0.222	0.081

This table shows the relationship between the amount of information provided by creators (word count in the description) and the amount raised by project category.

*Notes*: *t*-statistics are in parentheses; marginal effects are in italics.

*, **, and *** designate statistical significance at the 10%, 5%, and 1% levels, respectively. In all specifications, we control for *Goal*, *Duration*, *Duration*^*2*^, *Latitude*, *Longitude*, *Project number*, *Average number of projects in the same sub-category*, and *Edits*.

**Table 6:** Multivariate analysis of description length (word count censored at 99%) on *ln*(Backers).

	Art	Comics	Dance	Design	Fashion	Film & Video	Food	Games	Music	Photography	Publishing	Technology	Theatre
	(20)	(21)	(22)	(23)	(24)	(25)	(26)	(27)	(28)	(29)	(30)	(31)	(32)
Word count	.0022***	.0023***	.0021***	.0031***	.0029***	.0024***	.0022***	.0022***	.0026***	.0025***	.0022***	.0019***	.0019***
	(15.96)	(8.85)	(5.98)	(13.08)	(11.56)	(28.12)	(10.30)	(11.75)	(24.75)	(8.92)	(16.81)	(6.62)	(8.87)
Word count2	-8.0e-07***	-6.4e-07***	-7.7e-07***	-9.7e-07***	-8.7e-07***	-8.0e-07***	-7.6e-07***	-4.9e-07***	-1.0e-06***	-8.5e-07***	-8.3e-07***	-4.3e-07***	-7.4e-07***
	(-10.32)	(-4.82)	(-3.52)	(-8.10)	(-6.20)	(-16.56)	(-6.29)	(-6.17)	(-12.87)	(-4.98)	(-12.08)	(-3.31)	(-4.99)
N (Obs.)	6283	1994	1025	2924	2180	17678	2770	3596	16736	1951	7490	1527	3600
N (Cluster)	5785	1663	915	2623	2054	16186	2612	2982	15580	1832	6967	1428	3218
*R*^2^	0.106	0.186	0.102	0.178	0.170	0.141	0.129	0.297	0.113	0.127	0.123	0.201	0.083

This table shows the estimated relationship between the amount of information provided by the creator (word count in the description) and the number of backers, by project category.

*Notes*: *t*-statistics are in parentheses; marginal effects are in italics.

*, **, and *** designate statistical significance at the 10%, 5%, and 1% levels, respectively. In all specifications, we control for *Goal*, *Duration*, *Duration*^*2*^, *Latitude*, *Longitude*, *Project number*, *Average number of projects in the same sub-category*, and *Edits*.

## Discussion

To assess the influence of information quantity on funding success, we measure the former as the word count of the project description and quantify the latter as the funds contributed and the number of contributing backers. Using these variables, we are able to demonstrate a clear non-linear relation between the amount of descriptive text and positive funding outcome, with a positive effect elicited in the lower word count range by increasing the number of words. We also demonstrate that, as evidenced by the inverted U-shape in Figs 3 and 4, in all Kickstarter product categories, there is an optimal number of words beyond which the project creator’s ability to attract contributions and contributors (backers) is reduced. As each category’s turning point varies, it may indicate a degree of flexibility within backer perceptions of what is considered to be too much information, where the consumption of information is more elastic. For example, the categories of *Games* and *Publishing* both attempt to capture the imagination of their respective recipients. Yet, the optimal length of the project description is greater for *Games* than that of *Publishing*. It may be that in order to sell the game you need to sell the story, whereas in publishing you need to sell the story without giving away the plot line, and thus need to be more succinct. Regardless of whether the creator is emphasizing certain points or providing extra detail, crossing this optimal point (which occurred in 2% of the cases analysed) has a negative impact on overall project success by deterring backers and their funds. This effect may be caused by the extra effort needed to read a lengthy text or by sheer information overload. On the other hand, given that projects with fewer words are more likely to be scams, much shorter texts may not provide sufficient detail to convince potential contributors of the project’s high quality or even its legitimacy [38].

In drawing these conclusions about the influence of text length cues on decision-making behaviour, we are careful to consider the limited cognitive capacity of bounded rational individuals. Nevertheless, our analysis is not without limitations. First, it is highly likely that we have an omitted variable bias. For example, social network sizes, the presence and scope of images and videos, the frequency and timing of updates, as well as spelling errors within the text can all significantly affect success [27], [39], [40], [41]. Nor can we control for the distinctive personal characteristics of backers, such as age or category interests, which may influence their funding decisions by shaping their information capacity or interest. Moreover, although we measure the quantitative aspect of the text, we do not operationalize its qualitative features, which thus offer a useful direction in which to extend the research. Finally, although we try to account for a suspected endogeneity bias, future studies might better address this aspect by working with more precise data and monitoring all changes over time.

## Supporting information

S1 Fig Effects of word count on *ln*(Raised) based on sub-category. * designates unspecified sub-categories. Each section is based on the sub-category that is offered on Kickstarter during the time period of data collection, and represents the marginal effects of the word count based on specification (3).
(TIF)

S2 Fig Effects of word count on *ln*(Backers) based on sub-category. * designates unspecified sub-categories. Each section is based on the sub-category that is offered on Kickstarter during the time period of data collection, and represents the marginal effects of the word count based on specification (6).
(TIF)

S1 Table Search terms to identify type of edit. *Note*: All terms are case sensitive unless the word/phrase contains at least one upper case letter.
(DOCX)
