# Supplementary figures and images for: How much is too much? The effects of information quantity on crowdfunding performance
Source: PLoS One. 2018 Mar 14;13(3):e0192012. doi: 10.1371/journal.pone.0192012 (PMC5851530; doi:10.1371/journal.pone.0192012)

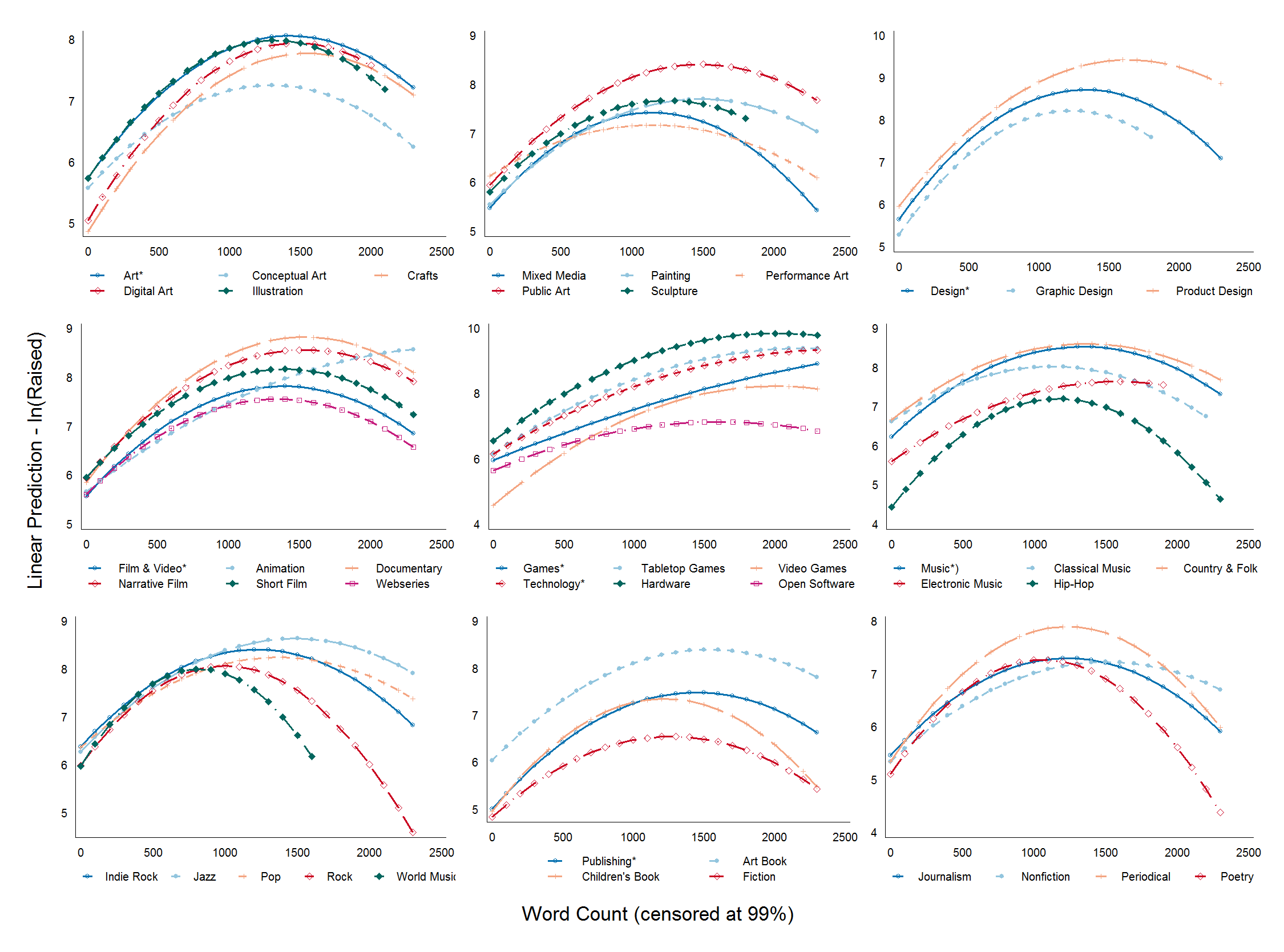

Supplement: S1 Fig — * designates unspecified sub-categories. Each section is based on the sub-category that is offered on Kickstarter during the time period of data collection, and represents the marginal effects of the word count based on specification (3). (TIF) [file pone.0192012.s001.tif]

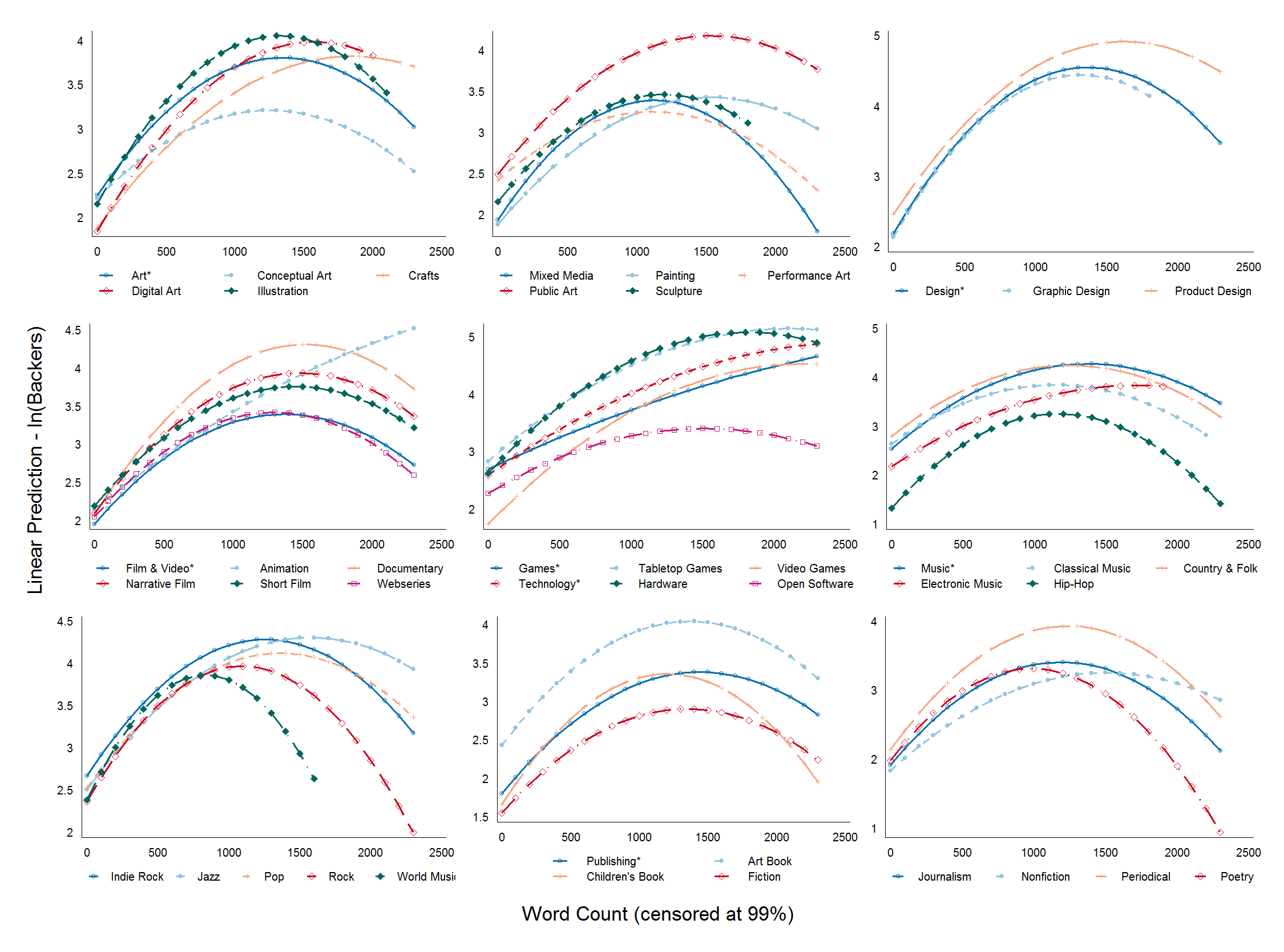

Supplement: S2 Fig — * designates unspecified sub-categories. Each section is based on the sub-category that is offered on Kickstarter during the time period of data collection, and represents the marginal effects of the word count based on specification (6). (TIF) [file pone.0192012.s002.tif]
