# Supplementary material for: How much is too much? The effects of information quantity on crowdfunding performance
Source: PLoS One. 2018 Mar 14;13(3):e0192012. doi: 10.1371/journal.pone.0192012 (PMC5851530; doi:10.1371/journal.pone.0192012)
Supplement: S1 Table — Note: All terms are case sensitive unless the word/phrase contains at least one upper case letter. (DOCX) [file pone.0192012.s003.docx]

| General edit | Reminder | Progress |
| --- | --- | --- |
| Update* | days to go | *ve reached |
| UPDATE* | hours to go | we reached |
|  | weeks to go | goal reached! |
|  | days left | GOAL REACHED |
|  | hours left | goal met! |
|  | weeks left | GOAL MET |
|  | days remain | reached our goal |
|  | hours remain | we did it! |
|  | weeks remain | we made it! |
|  |  | WE DID IT |
|  |  | WE MADE IT |
|  |  | funding reached |
|  |  | we are funded! |
|  |  | WE ARE FUNDED |
|  |  | e are FUNDED |
|  |  | has been reached |
